# Evaluation of Oxidative Stability and Quality Parameters of Sunflower Oil Enriched With Different Levels of Retinyl Palmitate Under Different Storage Conditions

**DOI:** 10.1002/fsn3.70463

**Published:** 2025-06-18

**Authors:** Gülsüm Şahin Bodur, Alev Keser, Merve Akpinar Uzun, Aziz Tekin

**Affiliations:** ^1^ Department of Nutrition and Dietetics, Faculty of Health Sciences Cankiri Karatekin University Cankiri Turkiye; ^2^ Department of Nutrition and Dietetics, Faculty of Health Sciences Ankara University Ankara Turkiye; ^3^ Department of Food Engineering, Faculty of Engineering Ankara University Ankara Turkiye

**Keywords:** oxidative stability, retinyl palmitate fortification, storage conditions, sunflower oil

## Abstract

This study aimed to evaluate the oxidative stability and vitamin A retention of fortified sunflower oil intended for edible use, under different storage conditions over a six‐month period. Sunflower oil samples were enriched with retinyl palmitate in four concentrations (15,30,60,90 μg/g) and stored in transparent and brown glass bottles under daylight and dark conditions for six months. Oxidative stabilities of the samples (Rancimat) were measured at the beginning of the study. The other analysis including retinyl palmitate levels, peroxide value, conjugated diene and triene, and free fatty acids contents were performed at the beginning and monthly intervals over a six‐month storage period. The results were evaluated using some statistical tests at significance levels of *p* < 0.05 and *p* < 0.001. The analysis revealed that, at the end of the sixth month, the highest levels of retinyl palmitate were retained in the groups fortified with 90 μg/g and stored in dark conditions, specifically in transparent bottles (89.39 ± 1.38 μg/g) and brown bottles (80.87 ± 4.05 μg/g). The highest peroxide values were observed after storage in the control group stored under light conditions in transparent bottles (43.92 ± 4.65 meq O_2_/kg oil), and in the group fortified with 15 μg/g under the same conditions (55.66 ± 14.12 meq O_2_/kg oil). Enriched sunflower oil exhibited a statistically significant increase in free fatty acid contents over time. In conclusion, storage time, light exposure, and bottle color significantly influenced oxidation and quality parameters. Proper storage conditions, particularly protection from light, are essential for maintaining the stability of retinyl palmitate‐enriched sunflower oil. Based on these findings, it is recommended that vitamin A‐enriched sunflower oils be stored in brown or light‐resistant bottles, kept away from direct light, and consumed relatively promptly to minimize oxidative degradation and preserve vitamin A stability.

## Graphical abstract

This study highlights the importance of storage conditions in maintaining the stability of retinyl palmitate‐enriched products, sunflower oil. Light exposure accelerates degradation; therefore, storing the oil in dark, light‐resistant bottles is essential. Additionally, higher concentrations of retinyl palmitate provided greater oxidative stability.

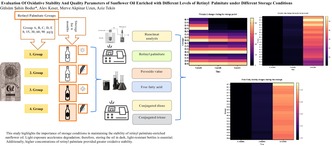

## 1 Introduction

Vitamin A deficiency (VAD) is the third most significant micronutrient deficiency affecting global public health. According to the World Health Organization (WHO), approximately 2.8 million preschool‐aged children are at risk of blindness due to VAD, and the health of an estimated 251 million individuals is adversely affected worldwide (WHO 2023). In Turkey, a regression‐based analysis conducted by WHO estimated the prevalence of VAD among preschool children to be 12.4% (WHO 2023), whereas another study reported the rate to be 14.7% among healthy children (Ekemen et al. 2018). These figures highlight the urgent need for effective and sustainable strategies to combat VAD.

In many developing countries, fortification of edible oils with vitamin A compounds such as retinyl palmitate, retinyl acetate, and β‐carotene has been mandated to reduce the burden of VAD. Fortification levels vary by country, with edible oils fortified at 1.5–3.0 mg/100 g in Bangladesh, 11 μg/g in India, and 18.15 μg/g in Pakistan (Hasan et al. 2023). However, despite the growing importance of food fortification, there is a notable scarcity of studies investigating the stability and behavior of vitamin A compounds in fortified sunflower oil, particularly under long‐term storage conditions (Saad et al. 2017; Walters et al. 2019).

Oxidation is a key determinant of the functionality and shelf life of oils in technological processes, and it negatively influences their color, taste, odor, and nutritional value (Maszewska et al. 2018). Sunflower oil exhibits high susceptibility to oxidation during storage, characterized by a rapid onset of peroxidation. Linoleic acid (60%–70%), a polyunsaturated fatty acid, and the limited content of γ‐ and δ‐tocopherol, which are known for their strong antioxidant activity, further contribute to the oil's sensitivity to oxidation (Pokorny et al. 2001). Interestingly, vitamin A itself is a sensitive yet biologically active compound that may participate in or mitigate oxidation processes. Retinyl esters, in particular, have been shown to form hydrogen bonds with oxidized lipids, thereby reducing peroxide formation and enhancing vitamin A bioavailability during storage (Allen et al. 2006).

Storage conditions—specifically temperature, light exposure, and packaging—are known to influence both oxidative degradation and vitamin A stability (Pignitter et al. 2014). However, comprehensive studies examining the combined effect of these factors over extended storage periods in vitamin A‐fortified sunflower oil remain limited. This study aims to fill this gap by quantitatively evaluating the oxidative stability and vitamin A retention of sunflower oil enriched with varying concentrations of retinyl palmitate, stored under different light and packaging conditions over a six‐month period. Unlike previous research that has typically focused on either oxidation or vitamin A stability in isolation, this study provides an integrated, time‐resolved analysis of both parameters. Furthermore, by simulating real‐world storage scenarios without the addition of synthetic antioxidants, this study offers novel insights into the practical formulation, packaging, and shelf‐life strategies for vitamin A‐fortified edible oils.

## 2 Material and Methods

### 2.1 Materials

In this study, refined sunflower oil was purchased from a local chain market with a high product turnover, while retinyl palmitate (Merck KGaA, Darmstadt, Germany) was procured internationally. Other chemicals used in the study, including acetic acid (Isolab), chloroform (Carlo Erba), ethanol (Isolab), sodium hydroxide (Tekkim), iso‐octane (Isolab), and hexane (Isolab), were obtained from a domestic supplier.

### 2.2 Sample Preparation

In this study, the concentrations of retinyl palmitate (0, 15, 30, 60, and 90 μg/g) were selected based on physiological relevance, regulatory guidelines, and existing literature. The daily dietary energy intake was assumed to be 2000 kcal, as suggested in the TUBER (2022) guidelines. Considering that approximately 30% of total energy should be derived from fat, this corresponds to 600 kcal from fat, or roughly 65 g of fat per day. Assuming that all dietary fat consumed is fortified, and taking into account that an average of 80% (range: 70%–95%) of fat is metabolically absorbed, the effective fat intake would be approximately 52 g. Given that the maximum tolerable intake level for vitamin A is 3000 μg/day (TUBER 2022), the calculated maximum permissible fortification level is approximately 58–60 μg/g. Therefore, in this study, two concentrations below this threshold (15 and 30 μg/g), one at the threshold (60 μg/g), and one slightly above (90 μg/g) were selected to evaluate the dose‐dependent stability, degradation, and safety profile of retinyl palmitate under various storage conditions.

International recommendations and previous research further support these selected concentrations. For example, edible oils are fortified with 15–30 μg/g of vitamin A in Bangladesh, 11 μg/g in India, and 18.5 μg/g in Pakistan (Begum et al. 2024; Indonesia Ministry of Health 2013; Rehman et al. 2013). The World Health Organization recommends a fortification level of approximately 33 μg/g for edible oils (WHO 2002). Moreover, Yokio Kakuda et al. evaluated the stability of vitamin A in fortified oils using three fortification ranges: 13.2–15.95 μg/g, 91.3–112.75 μg/g, and 167.75–191.95 μg/g, providing a comparative basis for selecting both moderate and high concentrations in the present study (WHO 2002).

In conclusion, the study analyzed sunflower oil samples enriched with retinyl palmitate at four concentrations (15, 30, 60, and 90 μg/g), along with a control group containing no retinyl palmitate. The experimental groups were given in Table 1. The batch mixing method was employed to enrich the sunflower oil in this study (Allen et al. 2006; Anonymous. 2020).

**TABLE 1:** Experimental groups based on retinyl palmitate concentration, light exposure, and bottle color.

Retinyl palmitate concentration (μg/g)	Light	Bottle color	Oil code
0	Light	Transparent	A‐1
	Light	Brown	A‐2
	Dark	Transparent	A‐3
	Dark	Brown	A‐4
15	Light	Transparent	B‐1
	Light	Brown	B‐2
	Dark	Transparent	B‐3
	Dark	Brown	B‐4
30	Light	Transparent	C‐1
	Light	Brown	C‐2
	Dark	Transparent	C‐3
	Dark	Brown	C‐4
60	Light	Transparent	D‐1
	Light	Brown	D‐2
	Dark	Transparent	D‐3
	Dark	Brown	D‐4
90	Light	Transparent	E‐1
	Light	Brown	E‐2
	Dark	Transparent	E‐3
	Dark	Brown	E‐4

### 2.3 Storage Conditions

The sunflower oils were placed in 200 mL transparent and brown‐colored glass bottles specifically designed for storage purposes in the laboratory. The bottles were stored at ambient temperature ranging from 26°C to 32°C on a laboratory bench exposed to daylight (light environment) and in a laboratory cabinet shielded from daylight (dark environment) for a period of six months. Analyzes were made with samples taken from these samples once a month. The temperature of each bottle was regularly monitored using a thermometer.

### 2.4 Analytical Methods

Rancimat, retinyl palmitate levels, peroxide value, conjugated diene, conjugated triene, and free fatty acids analyses of sunflower oil were conducted on the groups at the beginning of the study (month 0) and after enrichment. Subsequently, all analyses, except for Rancimat, were repeated monthly for a period of six months, with samples collected from sunflower oils. They are not ranked when analyzed.

The evaluation of resistance to autoxidation was carried out in accordance with the AOCS Official Method Cd 12–57, at 110°C using a Rancimat 743 (Metrohm AG, Herisau, Switzerland) under an air flow rate of 20 L/h. The induction period was recorded in hours (AOCS 1998).

Peroxide analysis was performed according to AOCS official method Cd 8–53 and results were expressed in meq O_2_/kg. Accordingly, a 3:2 solution of acetic acid: chloroform was added to 1 g of sample. Then 0.5 mL of potassium iodide (KI) solution was added to the mixture. After adding 30 mL of distilled water to dilute the mixture, 4–5 drops of starch solution were added to see the color change in the mixture. The mixture was then titrated with 0.1 N sodium thiosulphate. Record the amount of sodium thiosulphate used (AOCS 2003).

$$\text{Peroxide value}\;\left(\mathrm{meq}\;O_{2}/\mathrm{kg}\;\text{oil}\right)=\left(\left(S-B\right)*N^{*}1000\right)\backslash P$$

S: Sodium thiosulfate solution used (mL).

B: Sodium thiosulfate solution spent for the blank (mL).

N: Normality of the sodium thiosulfate solution used.

E: Sunflower oil quantity (g).

Free acidity was determined according to AOCS official method Ca 5a‐40. Free acidity was determined by titrating 5 g of fat dissolved in 35 mL of ethanol against 0,01 N sodium hydroxide (NaOH). To observe the color change, add 5–6 drops of phenolphthalein to the mixture before titration. The results are expressed as a percentage of oleic acid. The titration was terminated when a pink color appears. Record the amount of NaOH used (AOCS 2021).

$$\text{Free acidity}\;\left(\%\right):\left(\left(\;V_{\text{sample}}-V_{\text{blank}}\right)*N^{*}28,2\right)\backslash P$$

V: Amount of NaOH used (mL).

N: Normality of the NaOH solution used.

Conjugated diene and triene were analysed according to AOCS Official Method Ch5‐91. The specific absorption value at 232 nm increases with increasing conjugated diene formation, whereas the specific absorption value at 268 nm increases with increasing triene formation (AOCS 2017). The measurements were duplicated using a Shimadzu UV–VIS Spectrophotometer, Japan.

$$E=K\;\lambda =\mathrm{A\lambda}\backslash \left(c^{*}L\right)$$

Kλ = Specific absorption value at the read wavelength.

Aλ = Absorbance value at the wavelength read.

c = Concentration of the solution (g\100 mL).

L = Light path (1 cm).

Retinyl esters in fortified oil are determined by dissolving the oil in an organic solvent and reading the absorbance of the solution at 325 nm (Kamangar and Fawzi 1978; Subramanyam and Parrish 1976). In this study, vitamin A levels were measured using a UV–VIS spectrophotometer (Shimadzu, Japan).

$$\text{Retinyl palmitate}\;\left(\mathrm{mg}/\mathrm{kg}\right)=\left({{\mathrm{Abs}}_{\text{corrected}}}^{*}{\mathrm{Vf}}^{*}\text{CFspec}\right)/\left(a^{*}w\right)$$

Abs_corrected_ = Abs_oil_−Abs_unfortification oil_.

a: Absorption coefficient of retinyl palmitate in hexane (0.092).

Vf: Final volume (mL).

w: Sample weight (mg).

CFspec Spectrophotometer verification factor (ideally 1).

### 2.5 Statistical Evaluation of Data

The statistical analyses were performed using the Statistical Programme for Social Sciences (SPSS) version 25. Rancimat analysis was conducted in triplicate, whereas all other analyses were performed in duplicate. The mean and standard deviation values of the duplicate and triplicate measurements are reported. The Kruskal‐Wallis test was used to perform a comparative analysis of the initial Rancimat results. Subsequently, the Bonferroni test was applied to identify the specific group responsible for the observed significance. The dependent variables in this study consisted of quantitative measurements (e.g., peroxide value, retinyl palmitate level). The Four‐Way Repeated Measures ANOVA test was applied to find the difference between the groups at both aspects, and Bonferroni Post Hoc test was implemented for the pairs. A full‐factorial design (5 × 2 × 2) was employed in this study, consisting of five retinyl palmitate concentrations (0, 15, 30, 60, and 90 μg/g), two storage conditions (light and dark), and two bottle types (transparent and brown). This resulted in 20 experimental groups, each subjected to monthly analysis over six months. Data visualization was performed using the GraphPad programme (GraphPad Inc., La Jolla, CA, USA). For all statistical tests, the confidence interval was set at 95.0%, and results were evaluated at significance levels of *p* < 0.05 and *p* < 0.001.

## 3 Result Ans Discussion

### 3.1 Retention of Vitamin A

**FIGURE 1:**
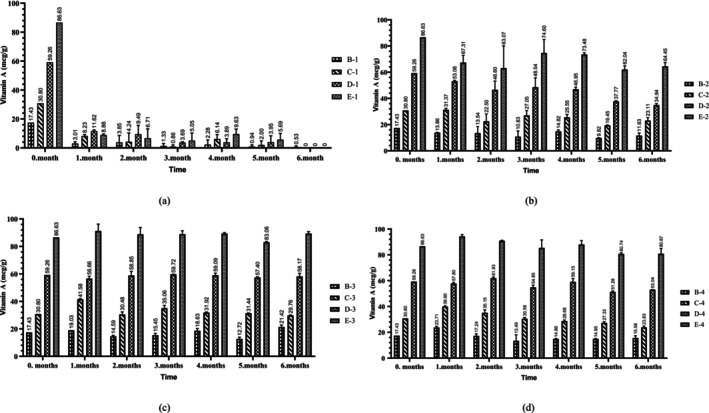
Retinyl palmitate variation (mcg/g) of sunflower oils with elevated retinyl palmitate content under different storage conditions. (a) light and transparent bottle (b) light and brown bottle (c) dark and transparent bottle (d) dark and brown bottle. Error bars represent standard deviation for each group at each time point.

In the present study, a total of five different retinyl palmitate groups were formed, including a control group that did not contain retinyl palmitate. Changes in the retinyl palmitate content of the prepared samples over the six‐month storage period are depicted in Figure 1. The analysis revealed that the highest levels of retinyl palmitate were observed in the E‐3 (89.39 ± 1.38 μg/g) and E‐4 (80.87 ± 4.05 μg/g) groups at the end of the sixth month of storage (Figure 1). Due to the presence of oxidative components in the oil, which can react with vitamin A, the protection rate was observed to increase with higher amounts of vitamin A added (Kidolezi 2018). Interestingly, vitamin A conservation levels were 103.1% and 93.3%, respectively, in these groups. Retinyl palmitate levels were found to be stable in the absence of light. Notably, it was observed that retinyl palmitate levels remained relatively stable across all samples stored in brown bottles, regardless of light exposure. Conversely, it was found that retinyl palmitate underwent rapid degradation when the samples were exposed to light. At the end of the sixth month of storage, vitamin A levels were reduced to zero in the C‐1, D‐1, and E‐1 groups stored under light conditions in transparent bottles (Figure 1). This decline was hypothesized to result from both environmental factors and bottle color. Additionally, the decrease in vitamin conservation associated with increasing oxidation is considered a contributing factor. This issue has also been investigated in some other studies. For instance, (Pignitter et al. 2014) demonstrated that the initial vitamin A concentration decreased by 84.8% when soybean oil fortified with retinyl palmitate (31.6 IU/g) was exposed to sunlight for 56 days at ambient temperature in closed, light‐permeable bottles (Pignitter et al. 2014). Similarly, in a separate study, sunflower oil fortified with 30–60 mg/L retinyl palmitate was exposed to solar radiation for a duration of 35 days in Malawi. This study revealed that vitamin A levels declined by 40% by the end of the experiment, while retinyl palmitate levels increased with extended storage periods (Ulemu et al. 2016). In this study, the primary effects of time, group, environment, and bottle color, as well as all double, triple, and quadruple interactions (*p* < 0.001), were found to have statistically significant effects on vitamin A levels (Table 2). As a result of post hoc tests, all pairwise comparisons between the groups were statistically significant (*p* < 0.001). However, there was a significant difference (*p* < 0.05) between the mean retinyl palmitate values at 0 month‐1 month, 1 month‐2 month and 4 month‐5 months for the time factor (data not shown).

**TABLE 2:** Four‐way ANOVA results for retinyl palmitate, peroxide value, and free fatty acid levels according to fortification level, storage condition, bottle color, and time.

Source	Retinyl palmitate			Peroxide value			Free fatty acids		
	MS	df	*p*	MS	df	*p*	MS	df	*p*
Group	30,156	3	0.000*	31	4	0.028**	0	4	0.000*
Environment	31,493	1	0.000*	740	1	0.000	0	1	0.000*
Bottle Color	10,180	1	0.000*	220	1	0.000	0	1	0.000*
Group×Environment	3359	3	0.000*	5	4	0.669	0	4	0.000*
Group×Bottle Color	1241	3	0.000*	1	4	0.929	5	4	0.000*
Environment×Bottle Color	12,414	1	0.000*	15	1	0.212	8	1	0.000*
Group×Environment×Bottle Color	1331	3	0.000*	2	4	0.869	0	4	0.000*
Error (between)	2393			1339			1805E‐5		
Time	1095	6	0.000*	6173	6	0.000	0,2	6	0.000*
Time×Group	111	18	0.000*	15	24	0.000	0	24	0.000*
Time×Environment	888	6	0.000*	182	6	0.000	0	6	0.000*
Time×Bottle Color	305	6	0.000*	51	6	0.000	0	6	0.000*
Time×Group×Environment	113	18	0.000*	7	24	0.065	7	24	0.000*
Time×Group×Bottle Color	39	18	0.000*	1	24	0.996	5	24	0.000*
Time×Environment×Bottle Color	393	6	0.000*	7	6	0.185	5	6	0.000*
Time×Group×Environment×Bottle Color	42	18	0.000*	3	24	0.936	4	24	0.000*
Error (within)	9395			5132			2354–6		

*Note:* Four‐way ANOVA test statistics, *p < 0.001 **p < 0.05.

Abbreviations: df, degrees of freedom; MS, mean squares.

### 3.2 Oxidative Stability and Quality Parameters

**FIGURE 2:**
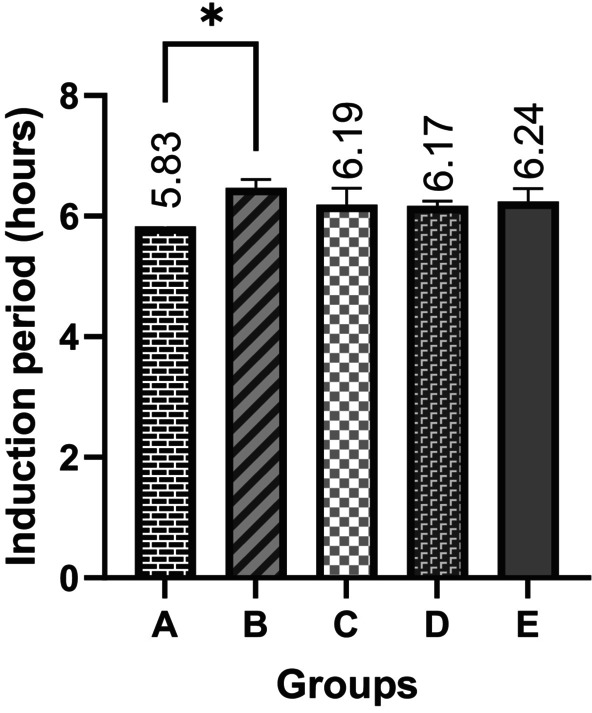
Induction periods of the sunflower oil samples fortified with different amounts of retinyl palmitate. *p < 0.05, Kruskal Wallis. Error bars represent standard deviation for each group at each time point.

In the present study, Rancimat, peroxide value, conjugated diene, and conjugated triene analyses were conducted to determine the oxidative stability of the oils. The induction periods of the samples measured by Rancimat were given in Figure 2. Induction period was shortest in group A (5.83 ± 0.14 h) and longest in group B (6.47 ± 0.14 h), which were control and 15 μg groups, respectively, and the difference between these groups was important (*p* < 0.05) (Figure 2). However, retinyl palmitate supplementation did not affect oxidative stability at levels of 30 μg and above. The variation in induction periods observed across studies may be attributed to differences in oil type, measurement parameters (e.g., temperature, air flow rate), or the amount of added antioxidant.

**FIGURE 3:**
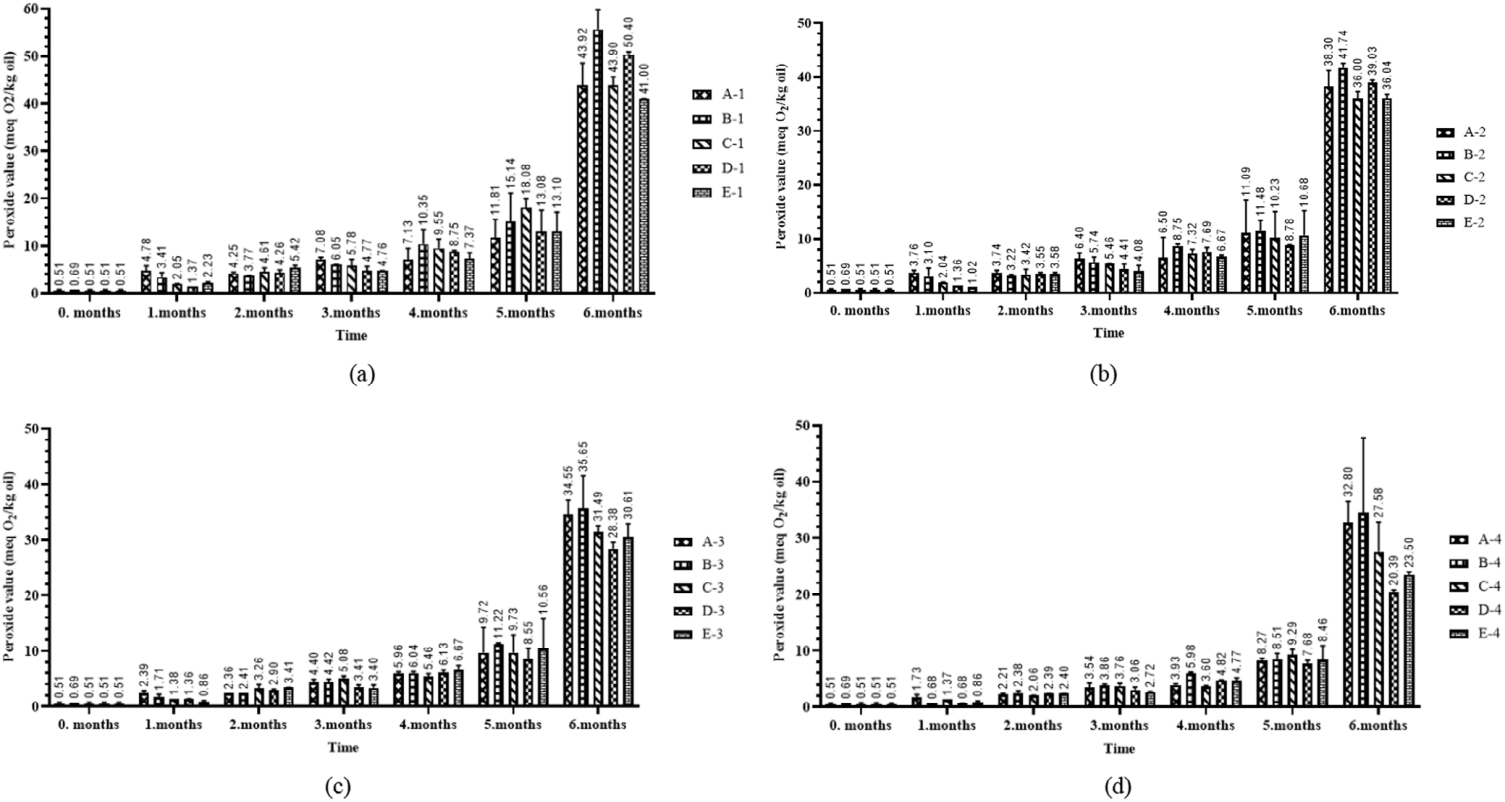
Peroxide values of sunflower oils with elevated retinyl palmitate content under different storage conditions: (a) light and transparent bottle, (b) light and brown bottle, (c) dark and transparent bottle, (d) dark and brown bottle.

In this study, peroxide values showed an upward trend, whereas conjugation values exhibited variability. It is well established that the peroxide value for vegetable oils should not exceed 10 meq O_2_/kg, a threshold reached after the fifth month of storage (Anonymous 2019; TGK 2012). Therefore, in fortified sunflower oils, exceeding this threshold after five months suggests a limited shelf life under the tested conditions, emphasizing the need for protective packaging and proper storage practices. From a public health and industry standpoint, this emphasizes the importance of incorporating oxidative stability data into formulation, expiration date determination, and consumer guidance strategies. Moreover, it highlights the potential need for reformulation or protective packaging to enhance the long‐term efficacy of vitamin A fortification programs. At the end of the sixth month, the highest peroxide values were observed in A‐1 and B‐1 (43.92 ± 4.65 and 55.66 ± 14.12 meq O_2_/kg oil), whereas the lowest values were found in D‐4 and E‐4 (20.39 ± 0.41 and 23.49 ± 0.48 meq O_2_/kg oil) groups (Figure 3). These results were possibly due to exposure of the samples with low retinyl palmitate levels to light. Consequently, the oxidation was accelerated and led to an increase the peroxide values. Conversely, under dark conditions and elevated retinyl palmitate levels, the opposite effect was observed. The findings of the present study align with previous research in this field (Guiotto et al. 2014; Nderitu et al. 2018). However, a statistically significant effect was identified for group (*p* < 0.05), environment (*p* < 0.001), and bottle color (*p* < 0.001) on peroxide value. Furthermore, the two‐way interactions between time and group (*p* < 0.001), time and environment (*p* < 0.001), and time and bottle color (*p* < 0.001) demonstrated statistically significant effects (Table 2). In addition, according to post hoc tests for statistically significant main effects, the difference between the groups was due to the 15 mcg‐90 mcg (*p* < 0.05) retinyl palmitate groups. For the time factor, pairwise comparisons of all consecutive months were statistically significant (*p* < 0.05) (data not shown).

**FIGURE 4:**
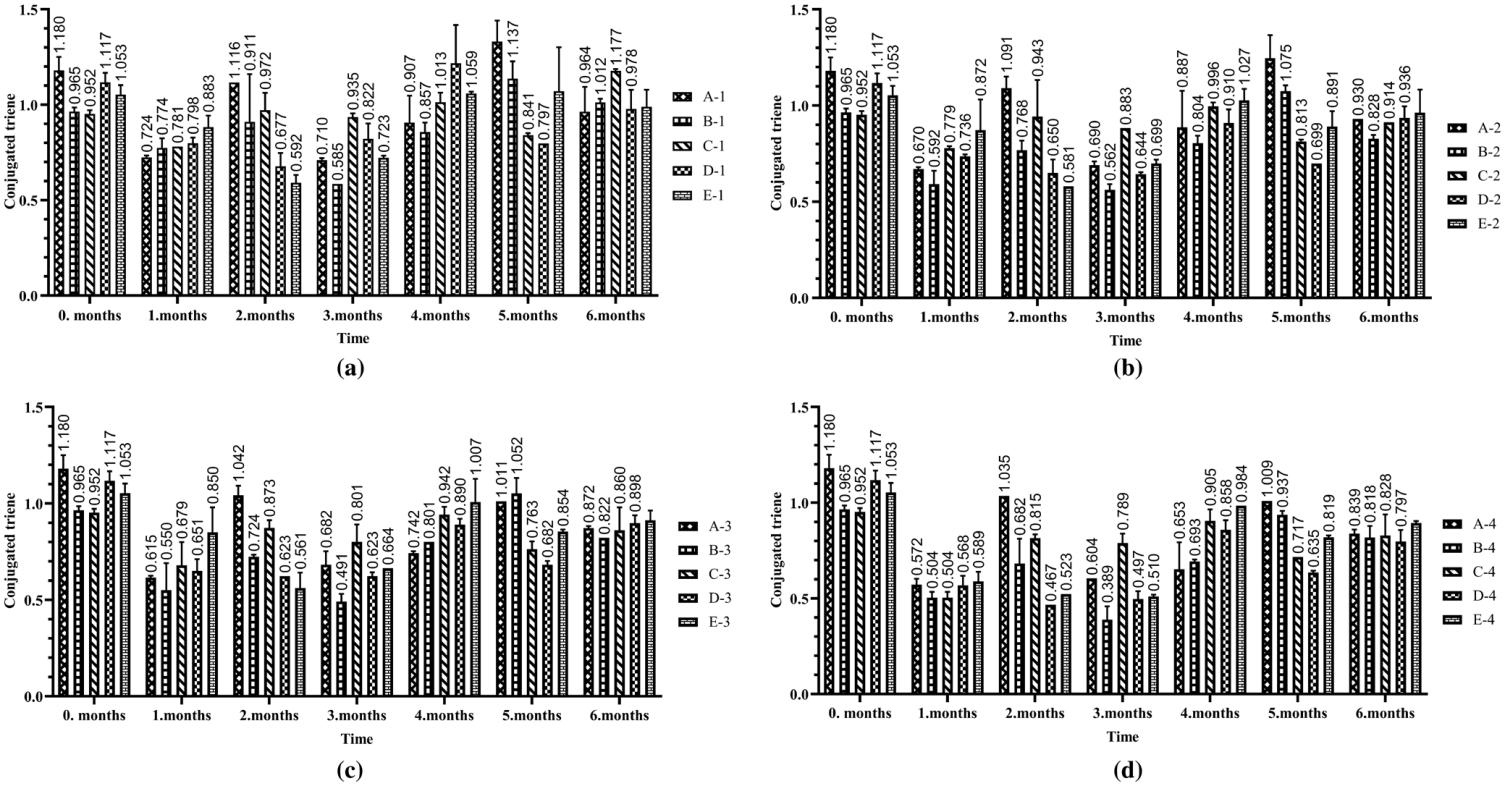
Conjugated triene variation of sunflower oils with elevated retinyl palmitate content under different storage conditions: (a) Light and transparent bottle; (b) light and brown bottle; (c) dark and transparent bottle; (d) dark and brown bottle.

**FIGURE 5:**
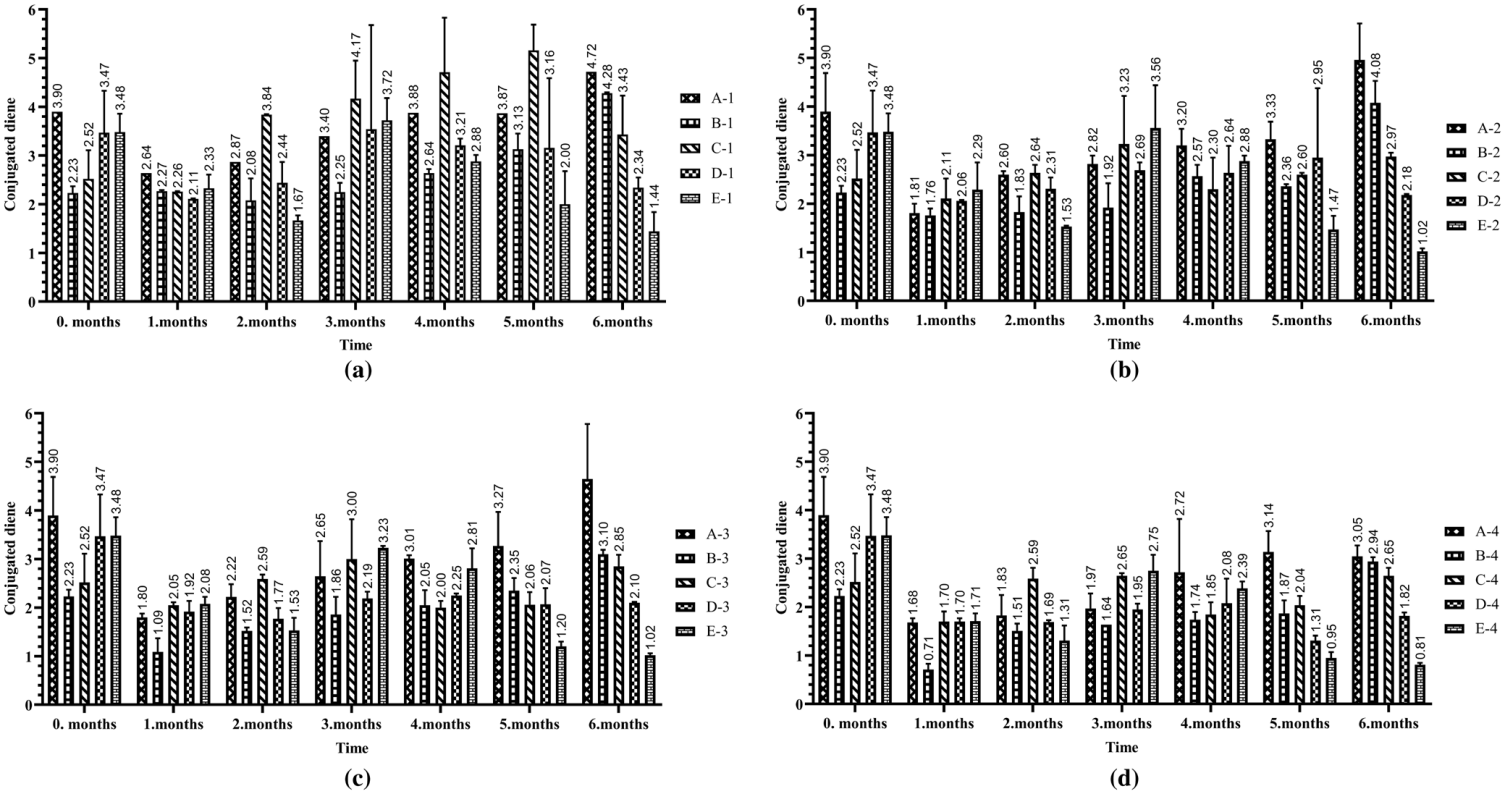
Conjugated diene variation of sunflower oils with elevated retinyl palmitate content under different storage conditions: (a) light and transparent bottle, (b) light and brown bottle, (c) dark and transparent bottle, (d) dark and brown bottle.

It was found that triene levels ranged between 0.95 ± 0.02 and 1.18 ± 0.07 during storage. The highest values at the end of the sixth month were observed in groups B‐1 (1.01 ± 0.02) and C‐1 (1.17 ± 0.01), whereas the lowest values were found in groups B‐4 (0.81 ± 0.06) and D‐4 (0.79 ± 0.06) (Figure 4). According to the results of post hoc tests, the difference between the groups was due to the difference between control and 15 mcg (*p* < 0.001), 30 mcg (*p* < 0.001), 60 mcg (*p* < 0.001), 90 mcg (*p* < 0.001); 15 mcg‐30 mcg (*p* < 0.001), 15 mcg‐90 mcg (*p* < 0.05), 30 mcg‐60 mcg (*p* < 0.001) dose groups. For the time factor, there was a significant difference (*p* < 0.05) between the mean conjugated triene values at 0 month‐1 month, 1 month‐2 month, 2 month‐3 month and 3 month‐4 month (data not shown). At the end of the sixth month, the highest levels of conjugated dienes were observed in groups A‐1 and A‐2 (7.72 ± 0.56 and 4.96 ± 1.75), whereas the lowest levels were recorded in the samples of E‐3 and E‐4 (1.02 ± 0.04 and 0.81 ± 0.04) (Figure 5). According to the results of post hoc tests, the difference between the groups was due to the difference between the dose groups of control‐15 mcg (*p* < 0.001), control‐30 mcg (*p* < 0.05), control‐60 mcg (*p* < 0.001), control‐90 mcg (*p* < 0.001), 15 mcg‐30 mcg (*p* < 0.001), 30 mcg‐60 mcg (*p* < 0.05), 30 mcg‐90 mcg (*p* < 0.001). When the time factor was analyzed, there was a significant difference (*p* < 0.05) between the mean conjugated diene values at 0 month‐1 month, 1 month‐2 month, 2 month‐3 month and 5 month‐6 months (data not shown). Changes in conjugated dienes and trienes are likely to be due to the formation of secondary oxidation products. However, the measurement method employed, the type of oil, and the antioxidant content may also influence these results. This conclusion was supported by some studies (Ulas 2015), although there are others reporting different findings (Al‐Dalain et al. 2011). Conjugated diene and conjugated triene values are UV spectrophotometric parameters widely used to detect primary and secondary oxidation products in edible oils. An increase in these values indicates that the oil has undergone oxidative degradation, which may result in unfavorable changes in flavor, odor, and nutritional profile. Likewise, elevated free fatty acid levels indicate hydrolytic degradation, which negatively impacts sensory characteristics and accelerates lipid oxidation. These parameters are essential quality indicators in the context of food applications, as they directly affect shelf life, consumer acceptability, and marketability of fortified oils.

**FIGURE 6:**
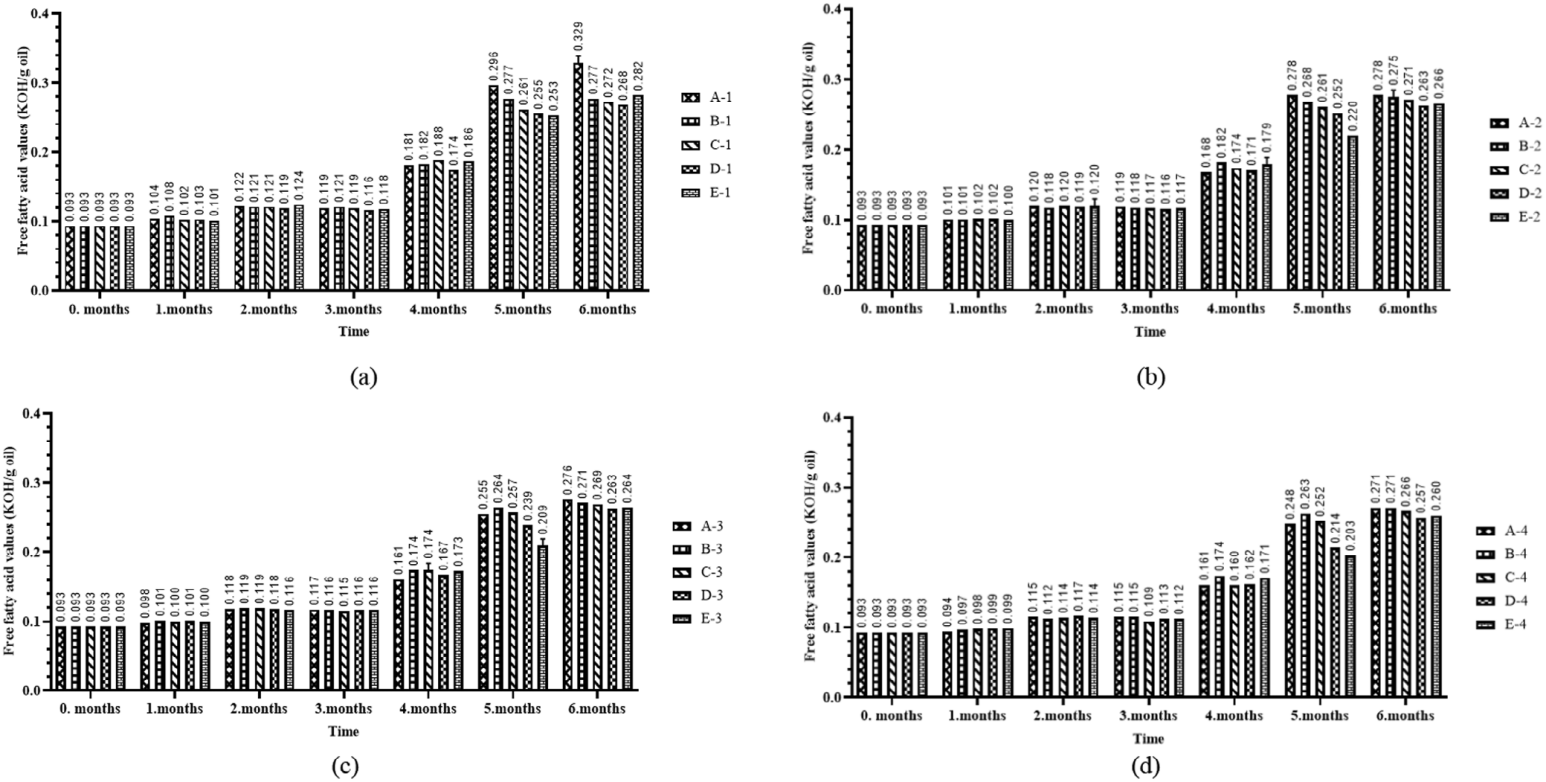
Free fatty acid values variation of sunflower oils with elevated retinyl palmitate content under different storage conditions: (a) light and transparent bottle; (b) light and brown bottle; (c) dark and transparent bottle; (d) dark and brown bottle.

In this study, free fatty acid percentage in the initial analyses averaged 0.093% which was below the maximum recommended value (0.3%) for the refined vegetable oils (Anonymous 2019). It was noted that the enrichment process did not affect the initial values. At the end of the sixth month, the highest values of free fatty acids were observed in groups A‐1 and E‐1 (0.329% ± 0.01% and 0.282% ± 0.00%), whereas the lowest values were recorded in groups D‐4 and E‐4 (0.257% ± 0.00% and 0.260% ± 0.00%) (Figure 6). As the four‐way interaction of time, group, environment, and bottle color on free fatty acid content (*p* < 0.001) was statistically significant, direct interaction terms were interpreted rather than main effects. The two‐way interactions of time and group (*p* < 0.001), time and medium (*p* < 0.001), time and bottle color (*p* < 0.001) and the three‐way interactions of time, group, and medium (*p* < 0.001), time, group, and bottle color (*p* < 0.001), time, medium, and bottle color (*p* < 0.001) were also significant on the free acidity value (Table 2). According to post hoc tests, the difference between groups was control‐30 mcg (*p* < 0.05), control‐60 mcg (*p* < 0.001), control‐90 mcg (*p* < 0.001), 15 mcg‐30 mcg (*p* < 0.001), 15 mcg‐60 mcg (*p* < 0.001), 15 mcg‐90 mcg (*p* < 0.001), 30 mcg‐60 mcg (*p* < 0.001), and 30 mcg‐90 mcg (*p* < 0.001) dose groups. For the time factor, consecutive pairwise comparisons of all months are statistically significant (*p* < 0.05) except for month 0–1 (data not shown). In this study, enriched sunflower oil exhibited a statistically significant increase in free fatty acid values over time during the storage process (Figure 6). These findings are supported by similar studies (Kucuk and Caner 2005; Nderitu et al. 2018), although some studies report contrasting results (Carelli et al. 2005; Hemery et al. 2015). This difference could be attributed to the amount of vitamins added, the variation in storage time, and the increase in oxidants resulting from prolonged exposure to storage conditions. However, the concentration of free fatty acids in vegetable oils is influenced by numerous factors, including the quality and variety of the raw material, harvesting conditions, processing and frying methods, storage conditions, and the time of oil production (Skiera et al. 2012).

### 3.3 Effect of Light Exposure

In this study, at the end of six months, vitamin A levels were reduced to zero in the samples (C‐1, D‐1, E‐1 groups) stored under light conditions in transparent bottles (Figure 1). However, the highest peroxide and free fatty acid values were observed in the samples subjected to similar storage conditions (Figures 3 and 6). Photooxidation, which accelerates oxidation in samples exposed to light, generates singlet oxygen—a form of oxygen that is more reactive than molecular oxygen and significantly increases the rate of oxidation (Caponio et al. 2005). Although vegetable oils are widely used in food fortification, they have disadvantages such as being highly susceptible to external factors such as photooxidation and having low oxidation stability.

### 3.4 Strengths and Limitations

This study has several strengths and limitations. A key strength of this study is that five groups, including the control group, were formed to determine the optimal level of retinyl palmitate for fortification. The inclusion of multiple oxidation parameters, such as Rancimat, peroxide value, and conjugated diene and triene, is important as it allows for cross‐validation in supporting the primary hypothesis. Free fatty acid, one of the quality parameters, was also analyzed. As recommended in previous studies, the use of an extended storage period is another strength of this study. However, one limitation is that antioxidant elements, such as tocopherol, which are naturally present in sunflower oil and can influence oxidation parameters, were not removed prior to analysis. One limitation of the present study is the exclusive use of glass containers, which may not fully reflect the diversity of packaging materials used in commercial settings. Future research should investigate the influence of different packaging types—especially commonly used plastics—on the oxidative stability and micronutrient retention in fortified oils. Although the results demonstrate the oxidative stability and chemical integrity of fortified sunflower oil, sensory attributes such as taste, odor, and mouthfeel were not evaluated. These aspects are essential for consumer acceptance and marketability. Future studies are planned to investigate the sensory properties of fortified oils in order to ensure both nutritional efficacy and consumer appeal.

## 4 Conclusion and Recommendations

Sunflower oil is highly prone to oxidative degradation during storage due to its high unsaturated fatty acid content, which can lead to off‐flavors, discoloration, reduced nutritional quality, and compromised safety. The forms of vitamin A used in food fortification not only provide nutritional benefits but may also contribute to improving oxidative stability.

In this study, the interactions between storage time, retinyl palmitate concentration, storage environment, and bottle color were shown to significantly affect the oxidative stability and quality parameters of enriched sunflower oil. Retinyl palmitate degradation was found to be accelerated under light‐exposed conditions, while higher fortification levels enhanced oxidative resistance. Based on these findings, it is recommended that vitamin A‐enriched sunflower oils be stored in brown or light‐resistant bottles, kept away from direct light, and consumed relatively promptly to minimize oxidative degradation and preserve vitamin A stability. Future research should investigate vitamin A retention under typical household cooking scenarios and extend the assessment to different types of edible oils and longer storage durations.

In practical applications, vitamin A‐fortified oils are best suited for cold consumption or low‐heat culinary use, as vitamin A is sensitive to thermal degradation at high cooking temperatures. Based on the findings of this study, several practical recommendations for industrial application can be made. Industries involved in the fortification of edible oils with vitamin A should utilize brown or light‐resistant packaging to minimize light‐induced degradation during storage and distribution. In addition, product labeling should provide clear instructions to consumers regarding storage away from light and heat. Considering the progressive loss of vitamin A over time, optimizing initial fortification levels based on expected storage durations could further help maintain nutritional quality until the end of shelf life. Although fortification improved oxidative stability within the initial months of storage, the cost‐effectiveness and technical sustainability of fortification strategies require further evaluation to ensure their practical applicability in real‐world conditions.

## Author Contributions

**Gülsüm Şahin Bodur:** conceptualization (lead), data curation (lead), formal analysis (lead), investigation (lead), methodology (equal), software (lead), writing – original draft (lead), writing – review and editing (lead). **Alev Keser:** conceptualization (equal), investigation (equal), methodology (equal), project administration (lead), supervision (lead), writing – review and editing (equal). **Merve Akpinar Uzun:** conceptualization (supporting), formal analysis (equal), investigation (equal), methodology (equal), supervision (equal), visualization (equal). **Aziz Tekin:** conceptualization (equal), formal analysis (equal), methodology (equal), resources (supporting), supervision (lead), writing – review and editing (equal).

## Ethics Statement

The authors have nothing to report.

## Conflicts of Interest

The authors declare no conflicts of interest.

## Data Availability

For access to the data utilized in this research, readers are encouraged to reach out to the corresponding author.
